# Determination of the Minimum Infusion Rate of Alfaxalone Combined with Electroacupuncture in Goats

**DOI:** 10.3390/ani11102989

**Published:** 2021-10-17

**Authors:** Lingling Liu, Mahmoud M. Abouelfetouh, Eman Salah, Rui Sun, Sha Nan, Mingxing Ding, Yi Ding

**Affiliations:** 1 College of Veterinary Medicine, Huazhong Agricultural University, No.1, Shizishan Street, Hongshan District, Wuhan 430070, China; liulingling678@163.com (L.L.); mahmoud.abouelfetouh@fvtm.bu.edu.eg (M.M.A.); eman.salah@fvtm.bu.edu.eg (E.S.); 15623277713@163.com (R.S.); nansha@webmail.hzau.edu.cn (S.N.); dmx@mail.hzau.edu.cn (M.D.); 2 Institute of Animal Science and Technology, Henan University of Animal Husbandry and Economy, Zhengzhou 450046, China; 3 Department of Surgery, Radiology and Anaesthesiology, Faculty of Veterinary Medicine, Benha University, Moshtohor 13736, Egypt; 4 Department of Pharmacology, Faculty of Veterinary Medicine, Benha University, Moshtohor 13736, Egypt

**Keywords:** alfaxalone, anesthesia, electroacupuncture, goat, minimum infusion rate

## Abstract

**Simple Summary:**

Goats have been used as animal models in research and are increasingly kept as pets like dogs and cats. Total intravenous anesthesia (TIVA) is increasingly used in companion animals. Electroacupuncture (EA) has been proven to produce analgesia, therefore, the objective of this study was to investigate the effect of EA on alfaxalone-based TIVA in goats. In this current study, the minimum infusion rate (MIR) of alfaxalone was determined in a combination with EA. The findings found that EA reduces the alfaxalone MIR required to prevent purposeful movement of the extremities in response to standardized noxious. In conclusion, EA provided analgesia, reduced the MIR of alfaxalone-based IV anesthesia, and thereby alleviated the adverse cardiorespiratory effects associated with alfaxalone anesthesia in goats.

**Abstract:**

Total intravenous anesthesia (TIVA) is increasingly used in companion animals. The effect of electroacupuncture (EA) on alfaxalone-based TIVA has not been previously reported in goats. Therefore, the objective of this study was to determine the minimum infusion rate (MIR) of alfaxalone required to prevent purposeful movement of the extremities in response to standardized noxious stimulation during its combination with EA in goats. Twelve clinically healthy goats weighing 18.5 ± 2 kg were randomly assigned to two groups (six goats/group). Alfaxalone alone (ALF group) and alfaxalone combined with EA (EA-ALF group). In the EA-ALF, alfaxalone was administered 30 min after EA stimulation. For induction of anesthesia, a bolus of alfaxalone was given at 3 mg/kg IV, and an infusion dose of 9.6 mg/kg/h was initially set for maintenance. The MIR of alfaxalone in both groups was determined by testing for responses to stimulation (clamping on a digit with Vulsellum forceps) at 10-min intervals after induction of anesthesia till the entire period of the experiment. Cardiopulmonary parameters and nociceptive threshold were measured throughout anesthesia. The median alfaxalone MIR was significantly lower in the EA-ALF group than the ALF group [9 (4.8–9.6) and 12 (11.4–18)], respectively; *p* = 0.0035). In the ALF group, goats anesthetized with MIR showed a significant increase in heart rate and cardiac output (*p* < 0.0001 and 0.0312, respectively), and decrease in respiratory rate (*p* < 0.0001), hemoglobin oxygen saturation (*p* = 0.0081), and rectal temperature (*p* = 0.0046) compared with those in the EA-ALF. Additionally, goats in the EA-ALF showed a higher nociceptive threshold than those in the ALF group (*p* < 0.0001). EA provided analgesia, reduced the MIR of alfaxalone-based IV anesthesia and thereby alleviated the adverse cardiorespiratory effects associated with alfaxalone anesthesia in goats.

## 1. Introduction

Total intravenous anesthesia (TIVA) is becoming a pivotal technique to achieve adequate depth of anesthesia in companion animals, such as dogs and cats [1,2]. TIVA is defined as a protocol of using an anesthetic agent as a constant rate infusion (CRI) alone or in a combination with premedication to provide hypnosis, antinociception, and reducing dosage requirements of anesthetic drugs, thus minimizing adverse effects [3]. The application of TIVA has not extensively been used in goats because of its possible complications in such species, including tympany, regurgitation, excessive salivation, and hypoxemia [4]. Therefore, there is limited information about TIVA protocols in goats.

Alfaxalone is a neuroactive steroid drug that is commonly applied for induction and maintenance of anesthesia in dogs [5] and cats [6]. Its action was mediated via intensifying the inhibitory effects of γ-aminobutyric acid (GABA) on GABAA receptors, resulting in nerve cell hyperpolarization, and blocking neural impulse transmission. Compared to other anesthetic agents, alfaxalone has several merits, including a broad margin of safety and fast onset of action, and less cumulative effects following repeated dosages [7]. Recently, alfaxalone has been reported to have minimal hemodynamic effects during anesthesia in goats [8,9,10] and sheep [11].

Electroacupuncture (EA) is the modern form of traditional hand acupuncture, where electrical stimulation is passed through acupuncture needles inserted at specific points in the skin. EA has been gaining more interest over the last decades, due to its several medical applications. EA could successfully enhance antinociception in several surgical interventions in humans [12] and domestic animals [13,14,15], without changing patient physiological variables. Additionally, the combination of EA and anesthetic drugs, referred to as EA-drug based anesthesia, was effectively used during surgeries in humans [16]. This combination improved the antinociceptive outcome, reduced the dosage requirement of the drug, thereby reducing the side effects. EA has been shown to substantially reduce the required dosage of anesthetics in humans [17], rats [18] and goats [19,20], by 40 to 46%, 50%, and >70%, respectively. In goats, the application of EA seems to produce safe analgesia compared to other commonly used injectable analgesics, such as alpha_2_ adrenergic agonists, which are associated with pulmonary adverse effects [4].

The use of EA combined with isoflurane anesthesia has been found to decrease the minimum alveolar concentration (MAC) of isoflurane in dogs without causing adverse hemodynamic effects [21,22]. In terms of injectable anesthetics, minimum infusion rate (MIR) can be used to determine the anesthetic dosage requirements during IV anesthesia. The MIR is comparable to the MAC of volatile anesthetics, which is recognized as the 50% effective dose (ED 50) of the anesthetic agent that allows 50% of subjects to not exhibit purposeful movement in response to noxious stimuli during IV anesthesia [23]. In goats, the feasibility of the application of EA with alfaxalone-based TIVA has not been previously reported. Therefore, the objective of this study was to determine the MIR of alfaxalone and the associated cardiorespiratory and antinociceptive effects during its combination with EA. Our hypothesis was that EA enhances anticonception and therefore reduces the MIR of alfaxalone.

## 2. Material and Methods

### 2.1. Animals

Yichang white goats (13- to 15-month-old and 18.5 ± 2 kg in body weight) were enrolled in this study. All goats underwent a comprehensive medical checkup including a complete blood, serum biochemical profile, and brucella melitensis detection test. Before the experiment, the animals were acclimatized to the experimental conditions for 1 h daily for one week. The animals were not allowed to get access to food for 16–20 h before the start of the experiment, but they had free access to water. This study was ratified by the ethical committee of Huazhong Agricultural University (HZAUGO-2019-005).

### 2.2. Experimental Design

This study used a prospective, randomized, blinded design. A total of twelve goats were randomly allocated to one of two equal-sized groups (ALF and EA-ALF groups; 6/each) using a computer program (www.randomizer.org) (accessed on 15 October 2019). In both ALF and EA-ALF groups, the anesthesia was induced and maintained with alfaxalone. Due to the induction duration of EA ranges from 25 to 30 min, the goats in the EA-ALF group received EA 30 min before getting anesthetized with alfaxalone. Cardiorespiratory and key echocardiographic parameters, nociceptive threshold of the flank, and tone of abdominal musculature were recorded before treatment (s) (baseline), immediately after induction of anesthesia, and 10 min after induction of anesthesia, and thereafter at 10-min intervals. To determine the MIR required to prevent purposeful movement in response to noxious stimuli, the CRI of alfaxalone was increased or decreased at 10 min after induction, and then at 10-min intervals. The baseline and experimental values were recorded while the goat was in a recumbent position.

#### 2.2.1. Electroacupuncture

The acupuncture regions were clipped and prepared aseptically with betadine antiseptic solution (10% povidone-iodine). EA stimulation was performed by a skilled acupuncturist using a set of insertion points including Bai hui (hundred meetings), San tai (3 platforms), Er gen (ear base), and San yan luo (3 Yang communication). The anatomic landmarks of these points have been reported for application in veterinary practice [20,24]. In the Bai hui point, a stainless-steel acupuncture needle (diameter, 0.30 mm; length, 100 mm) was perpendicularly inserted into the dorsal midline between the spinous processes of the last lumbar and the first sacral vertebrae to a depth of nearly 30 mm. In the San tai point, the needle was inserted cranioventrally into the dorsal midline between the spinous processes of the fourth and fifth thoracic vertebrae to a depth of approximately 40 to 50 mm. The Er gen points were located bilaterally ventrocaudal to the ear base at the depression between the ear base and cranial border of the transverse process of the atlas. The left Erh gen point was chosen in this study, in which the needle was inserted in the determined location till it reached the subcutaneous tissue of the left temporal fossa. In the San yan luo point, the needle was inserted ventromedially at approximately 30° angle 5 cm ventral to the lateral tuberosity of the radius in the groove between the common digital extensor and lateral digital extensor muscles of the left forelimb until reaching the subcutaneous tissue of the medial side. The needles were connected to their specific output ports of a multifunctional electric pulse generator (KWD-808I, Yingdi electronic medical equipment Co., LTD, Changzhou, Jiangsu Province, China). The frequency of EA was set at 60 Hz, and the intensity was held constant at 3.2 V for 30 min [25].

#### 2.2.2. General Anesthesia and MIR Determination

Before anesthesia, a 22G 2.5-cm catheter was inserted into each goat’s left cephalic vein. A single bolus of alfaxalone at a dose of 3 mg/kg [10] was administered over a period of 60 s for the induction with an additional dose of 1 mg/kg administered every 15 s, if needed, to facilitate tracheal intubation. A silicon endotracheal tube with 7.5 internal diameter was inserted by the aid of a 30-cm illuminated laryngoscope blade while the goats were placed into sternal recumbency. The goats were then positioned on the right lateral recumbency and allowed to breathe room air. Immediately after the induction, alfaxalone was initially infused at a dose rate of 0.2 mg kg/min [26] for the maintenance using an IV infusion pump (Volumetric infusion pump/controller, IMED Gemini PC-1 v8.13). The initial infusion rate was kept for an equilibration period of 10 min before testing nociception in response to noxious stimulation. The principal investigator was responsible for applying nociceptive tests. The MIR was determined through application of digit clamping of the non-dependent thoracic limb, in which Vulsellum forceps was closed on the soft upper part of a single digit of the hoof just below the coronary band for 60 s or until occurrence of a purposeful movement of the animal. The rate of alfaxalone infusion was modified according to the nociceptive responses. The positive response was strictly defined as gross movement of the head, trunk, or limbs. Tremors of the stimulated limb was not considered as a positive response. In the presence of a positive response, the alfaxalone infusion rate was increased by 0.02 mg/kg/min. Instead, if the response was negative, the rate was reduced by 0.02 mg/kg/min and maintained constant for another 10 min before delivering the subsequent stimulus. These adjustments were repeated until absence or presence of a purposeful response occurred. The alfaxalone MIR was calculated as the average of the infusion rates that permitted and prevented purposeful movement.

#### 2.2.3. Cardiorespiratory Parameters and Rectal Temperature

Heart rate (HR), non-invasive arterial blood pressure [systolic (SAP), diastolic (DAP) and mean (MAP)], respiratory rate (RR), hemoglobin oxygen saturation (SpO_2_), and rectal temperature (RT) were measured during anesthesia using a multiparameter electrocardiogram monitor (PM-9000 Express, Mindary Co., Ltd., Shenzhen, China) with a rectal temperature probe. SpO2 was recorded by placing the reflective pulse oximeter probe on the tongue of the goat. The blood pressure was measured with an appropriately sized cuff (width was about 40% of the circumference of the limb) wrapped around thigh of the left pelvic limb close to the inguinal groove to measure the pressure of the femoral artery [19].

#### 2.2.4. Echocardiographic Parameters

Two-dimensional guided M mode echocardiographic techniques was used for the cardiac imaging using an ultrasound machine (Siemens, X-300, Seongnam, Korea). The right parasternal long axis view was used to obtain the M mode images. To minimize overestimation, inaccuracies, and interobserver variability, an experienced echocardiographer was in charge of data collection and measurement of left ventricular (LV) internal dimensions from all M mode images. In this study, the recommendations from the American Society of Echocardiography and the European Association of Cardiovascular Imaging were used as a reference for the echocardiography and linear measurement of the LV internal dimensions during systole (LVIDs), diastole (LVIDd), the interventricular septum dimension (IVSD), and the left ventricular posterior wall dimension (LVPWD) [27]. An ultrasound integrated software was used to calibrate the LV internal dimensions, which automatically calculate ejection fraction (EF) (%), fractional shortening (FS) (%), and stroke volume (SV) (mL). Cardiac output (CO) (L/min) was calculated by multiplying the ECG-recorded HR by stroke volume (SV); thus, CO = HR * SV. The cardiac index (CI) (L/min/m^2^) was measured by dividing the CO by the animal’s body surface area (BSA) [28].

#### 2.2.5. Nociceptive Thresholds and Flank Muscle Tone

Potassium iontophoresis was used to measure the nociceptive threshold at the center of the left flank. This technique is applied over the skin and electrically elicit sensory nerve endings and nociception via inducing the gradual influx of potassium ions through the skin. The concentration of ions inflowed into the subcutaneous tissue was directly linked to the applied voltage or current [29]. The left flank was shaved, washed with soap and water, and degreased with 75% ethanol. Two electrodes were wrapped with gauze tape, soaked in a saturated solution of potassium chloride, and then applied 1–2 cm apart on the skin. A modified peripheral nerve stimulator (Direct Current Induction Therapy Apparatus; Shantou Medical Equipment Factory Co., Ltd., Shantou, China) was used to deliver a pulsed direct current through the electrodes. The voltage was increased stepwise to the level at which there were obvious tremors of the local skin and muscles and/or the animal attempted to turn its head towards the flank. At that point, the current was discontinued, and the volt level was recorded. To assess muscle tone, an investigator who was blinded to the delivered drug scored the degree of muscle relaxation during the experiment (Appendix A) [10]. Normal resting tone was taken a score of 2; less tone was represented by scores less than 2, and high tone was indicated by scores greater than 2.

### 2.3. Statistical Analysis

Statistical tests were performed using GraphPad Prism software version 8.0 (GraphPad Inc, San Diego, CA, USA). Continuous data (cardiorespiratory, echocardiographic parameters, and nociceptive threshold) were reported as the mean ± SD and induction doses, MIRs, MIR determination times, and flank muscle tone scores were presented as the median and range. Kolmogorov–Smirnov test was used to assess the normality (Gaussian distribution) of variables. After confirming a normal distribution of data using the Kolmogorov–Smirnov test. Two-way repeated measures ANOVA with Bonferroni’s multiple comparisons test were used to compare variables within each group and between groups. Unpaired t-tests were used to compare the induction doses and MIRs between groups, A *p* value < 0.05 was considered to be statistically significant.

## 3. Results

### 3.1. Effect of EA on Alfaxalone MIR

The doses of alfaxalone required for induction and maintenance of anesthesia with alfaxalone alone or combined with EA in goats are summarized in Table 1. There were non-significant differences in the alfaxalone dose required for induction of anesthesia between the ALF and EA-ALF group. Over the initial bolus of 3 mg/kg, additional boli of alfaxalone were required to complete the induction and achieve successful intubation in both groups. In comparison with the ALF group, the median alfaxalone MIR was significantly lower in the EA-ALF group (*p* = 0.0035). The time taken to determine the alfaxalone MIR was significantly longer in the ALF than the EA-ALF group (*p* = 0.0403).

### 3.2. Effects of Anesthesia Maintenance with Alfaxalone Alone or Combined with EA Cardiorespiratory Parameters

There were statistically significant differences found regarding cardiorespiratory parameters within and between the ALF and EA-ALF groups (Table 2). Compared to the baseline, HR increased significantly and *f*_R_ decreased significantly at two and ten minutes after induction of anesthesia and at alfaxalone MIR time in the ALF group (*p* < 0.05). In the EA-ALF, HR exhibited a significant increase at ten minutes after induction of anesthesia and at alfaxalone MIR time (*p* < 0.0001) with non-significant changes occurred in *f*_R_ compared to the baseline in all but ten minutes after induction of anesthesia (*p* = 0364). In both groups, SAP was significantly decreased (*p* < 0.05) at two and ten minutes after induction of anesthesia compared to the baseline. Additionally, DAP decreased with statistically significant differences detected at two and ten minutes after induction of anesthesia in the ALF group (*p* = 0.0396 and 0.0126, respectively), and at ten minutes after induction of anesthesia in the EA-ALF group (*p* = 0.396).

In the ALF group, SpO_2_ values being decreased significantly (< 90%) was observed at two minutes after induction of anesthesia and at the alfaxalone MIR (*p* = 0.0405) compared to the baseline. However, in the EA-ALF group, non-significant changes occurred in SpO_2_ and still maintained within the clinically acceptable range (94 to 97%). RT was significantly decreased during alfaxalone anesthesia at ten minutes after induction of anesthesia in the ALF group (*p* = 0.0210) and at alfaxalone MIR time compared to the baseline in both ALF and EA-ALF groups (*p* < 0.0001 and *p* = 0.0353, respectively). Compared with the ALF group, the EA-ALF showed a significant decrease in HR ten minutes after induction of anesthesia (*p* < 0.0001). Furthermore, the ALF showed a significant decrease in *f*_R_ at two minutes after induction of anesthesia and at alfaxalone MIR time (*p* = 0.0045, *p* < 0.0001) and RT at ten minutes after induction of anesthesia and at alfaxalone MIR time (*p* = 0.0210 and 0.0002) compared to the EA-ALF group.

### 3.3. Echocardiographic Parameters

In both groups, EF and FS fluctuated within the clinically acceptable range, with non-significant differences detected within and between groups (Table 3). In the EA-ALF group, CO and CI increased significantly at alfaxalone MIR (*p* = 0.0312 and 0.0354, respectively) compared to the baseline. In the ALF group, SV showed a significant decrease (0.0195) compared to the baseline. Compared with the ALF group, a significant increase in SV was observed at two and ten minutes after induction of anesthesia and at alfaxalone MIR time in the EA-ALF (*p* = 0.0005, 0.0015, and 0.0003, respectively). As well, CI was significantly increased at alfaxalone MIR time (*p* = 0.0451).

### 3.4. Nociceptive Thresholds and Flank Muscle Tone

In the ALF group, nociceptive threshold increased significantly at ten minutes after induction of anesthesia (*p* = 0.0093) compared to the baseline. However, in the EA-ALFX, the nociceptive threshold increased significantly at two and ten minutes and at alfaxalone MIR time (*p* < 0.0001). Compared with the ALF group, the EA-ALF showed a significant increase in the nociceptive threshold at two and ten minutes after induction of anesthesia and at the alfaxalone MIR time (*p* < 0.05). Flank muscle tone scores decreased with statistically significant differences (*p* = 0.0456) were detected at two minutes after induction of anesthesia in both the ALF group and EA-ALF group (Table 4).

## 4. Discussion

In this current study, the induction dose of alfaxalone required for induction of anesthesia was similar in both ALF and EA-ALF groups. However, the alfaxalone MIR required to prevent purposeful movement in response to noxious stimuli decreased significantly in the EA-ALF group in comparison with the ALF group (9 (4.8–9.6) and 12 (11.4–18), respectively; *p* = 0.0035), indicating the dose sparing effect of EA on alfaxalone anesthesia in goats. In prior reports, the effects of fentanyl or midazolam on MIR of alfaxalone were investigated in goats [26,30]. CRI of fentanyl (0.005 mg/kg/h) and midazolam (0.1 and 0.3 mg/kg/h) reduced the alfaxalone MIR by 30% in comparison to MIR for alfaxalone alone in goats. This reduction is nearly equivalent to that reported in our study. Therefore, the effect of EA is comparable to the alfaxalone sparing effects of fentanyl and midazolam in goats. EA has been widely used as analgesia in acute and chronic pain and is usually incorporated with other analgesics or anesthetics. EA has an adjunctive role in achieving analgesia and reducing the dose of dexmedetomidine and xylazine by 75% in goats [19,20]. The efficacy of EA combined with general anesthesia has been evaluated in patients undergoing cardiac surgery. EA has been found to reduce the dosage of intraoperative anesthetic drugs and in turn less complications and a quicker recovery [31]. Moreover, a combination of EA with epidural anesthesia resulted in 40 to 46% reduction in the dose of lidocaine required for performing abdominal surgery [17].

In this current study, goats in the ALF group exhibited a significant increase in HR compared with those in the EA-ALF group. This finding is in accordance with the effect of alfaxalone CRI reported in goats [32], sheep [11], and dogs [7]. The increase in HR may have been attributable to the excitatory effect of alfaxalone on sympathetic tone [33] or as a compensatory reflex to hypotension. Modulating the effect of alfaxalone on HR by EA might be due to augmentation of vagal activity and suppression of the sympathetic outflow [34]. Moreover, EA could induce the release of dynorphin, an endogenous neuropeptide, which interacts with the pre-synaptic kappa opioid receptors resulting in inhibition of noradrenaline to be released from the sympathetic neurons located in the sinus node [35,36]. A significant reduction in *f*_R_ and SpO_2_ was found in goats in the ALF group compared to those in the EA-ALF group. In this study, hypoventilation is mostly the cause of hypoxemia in goats. Alfaxalone has been reported to produce a dose-dependent respiratory depression in dogs [37], cats [38], and horses [39]. Therefore, the dose sparing effect of EA on the alfaxalone MIR could be the possible explanation for the absence of hypoxemia in the EA-ALF group.

The decrease in RT during anesthesia could be attributable to a depression in the thermoregulatory centers and a decrease in the skeletal muscle tone and metabolic activity [40]. EA corrected the respiratory depressant effect and hypothermia associated with alfaxalone anesthesia in goats; however, the mechanism of this action is still unclear. In this current study, the SpO_2_ values were lower in the ALF in comparison to the EA-ALF group.

In both ALF and EA-ALF groups, echocardiographic parameters fluctuated within the clinically acceptable with insignificant differences in SV and CO reported at alfaxalone MIR time. Prior studies revealed insignificant changes in CO, CI, and SV during alfaxalone anesthesia in goats [10], horses [41], and dogs [42,43]. In this current study, the alfaxalone-induced tachycardia is most likely the compensatory reflex for maintaining CO within the normal level range throughout the experiment [10,44].

The EA-induced antinociception is mainly affected by two factors, including the proper selection of acupuncture points and frequencies. The stimulation of the set of Bai hui, San tai, Erh gen, and San yan luo acupoints with frequencies between 30 and 100 Hz and intensity between 1 to 3.25 V has been substantiated to provide effective antinociception for abdominal surgical interventions in ruminants [45]. In this current study, EA setting at 60 Hz frequency was used to stimulate Bai hui, San tai, Erh gen, and San yan luo acupoints. This frequency of EA has been proven to provide an effective antinociceptive effect in goats [25]. The degree of EA-induced antinociception was assessed by potassium iontophoresis. Potassium iontophoresis provided a tool for determining changes in the nociceptive threshold using a reliable experimental pain stimulus that can be presented rapidly and repeatedly with minimal loss in consistency of a subject’s reported pain level [46]. In this current study, goats anesthetized with EA and alfaxalone combination showed a significant increase in the nociceptive threshold than those anesthetized with alfaxalone alone, indicating the synergistic antinociceptive effect of EA. The release of endogenous opioids as enkephalin, ẞ-endorphin, and dynorphin in CNS has mainly been implicated in the EA-induced antinociception [25,47].

In this current study, there were several limitations. Firstly, the sample size was small as this was the number of animals available to the investigators. Secondly, an invasive blood pressure method and blood gas analysis may provide a better assessment of the cardiopulmonary status during anesthesia. Thirdly, electrical stimulation used to measure the nociceptive threshold could stimulate all nerve terminals, not just those related to pain sensation and could be a confounding factor in assessing pain threshold.

## 5. Conclusions

This research focused on determining MIR of alfaxalone during its combination with EA in goats. EA provided antinociception, reduced the MIR of alfaxalone, and thereby modulated the cardiopulmonary side effects of alfaxalone-based TIVA in goats.

## Figures and Tables

**Table 1:** General anesthetic induction dose and minimum infusion rate (MIR) of alfaxalone required to prevent purposeful movement in response to noxious stimuli during anesthesia maintenance with alfaxalone alone (9.6 mg/kg/h) (ALF group) or with alfaxalone (9.6 mg/kg/h) after electroacupuncture induction (EA-ALF group).

Parameter	ALF Group (*n* = 6)	EA-ALF (*n* = 6)	*p* Value
Alfaxalone induction dose (mg/kg)	4 (3.3–4.4)	3.9 (3.4–4.5)	
Alfaxalone MIR (mg/kg/h)	12 (11.4–18)	9 (4.8–9.6) *	0.0035
MIR determination time (min)	50 (40–70)	30 (30–50) *	0.0403

* Significant difference between the two groups (*p* < 0.05). Data were expressed as median (range).

**Table 2:** Cardiorespiratory parameters and rectal temperature measured in goats at baseline and at 2, 10 min, and time at which alfaxalone infusion last prevented purposeful movement (t-MIR) during anesthesia maintenance with alfaxalone alone (9.6 mg/kg/h) (ALF group) or with alfaxalone (9.6 mg/kg/h) after electroacupuncture induction (EA-ALF group).

Parameter	Group	Time Points			
		Baseline	2 min	10 min	t-MIR
HR (beats/min)	ALF	93.8 ± 9.9	122.6 ± 17.4 *	147.5 ± 26.8 *^,^†	143.6 ± 23.2 *
			*p* = 0.0009	*^,^† *p* < 0.0001	*p* < 0.0001
	EA-ALF	98.1 ± 6.6	106 ± 16.3	112 ± 18.5 *	127.5 ± 12.3 *
				*p* < 0.0001	*p* < 0.0001
SAP (mmHg)	ALF	114 ± 5.2	91.5 ± 5.8 *	86.5 ± 6.4 *	111.3 ± 6.5
			*p* = 0.0002	*p* < 0.0001	
	EA-ALF	110.1 ± 6.9	91.1 ± 7.2 *	92.6 ± 4.4 *	111.5 ± 2.5
			*p* = 0.0011	*p* = 0.0026	
MAP (mmHg)	ALF	82.3 ± 11.7	68.8 ± 17.5	79 ± 15.9	89.5 ± 15.3
	EA-ALF	95.1 ± 9.4	72.2 ± 8.9	82.6 ± 4.3	91.6 ± 18.3
DAP (mmHg)	ALF	71.1 ± 10.1	54 ± 5.2 *	51.5 ± 8.7 *	81.1 ± 7.8
			*p* = 0.0396	*p* = 0.0126	
	EA-ALF	73.8 ± 8.9	61.1 ± 13.9	56.6 ± 9.1 *	75.1 ± 13.2
				*p* = 0.0396	
*f*_R_ (breaths/min)	ALF	20.5 ± 2.2	14 ± 3.5 *,†	15.1 ± 3.7 *	11.5 ± 2.3 *^,^†
			* *p* = 0.0020	*p* = 0.0133	* *p* < 0.0001
			† *p* = 0.0045		† *p* <0.0001
	EA-ALF	22.8 ± 3	20 ± 2.9	19 ± 3.1 *	21 ± 2.9
				*p* = 0.0364	
SpO_2_ (%)	ALF	95.1 ± 1.7	86.5 ± 6.4 *	92 ± 2.2	86.5 ± 5.5 *
			*p* = 0.0405		*p* = 0.0405
	EA-ALF	94.6 ± 1.9	94.6 ± 3.2	94.8 ± 2.1	94.1 ± 2
RT (°C)	ALF	39.1 ± 0.26	38.8 ± 0.45	38.6 ± 0.39 †	37.8 ± 0.41 *
				*p* = 0.0210	* *p* < 0.0001
					† *p* = 0.0002
	EA-ALF	39.2 ± 0.25	39.3 ± 0.37	39.2 ± 0.47	38.7 ± 0.24 *
					*p* = 0.0353

Heart rate (HR), systolic arterial blood pressure (SAP), mean arterial pressure (MAP), diastolic arterial blood pressure (DAP), respiratory rate (*f*_R_), hemoglobin oxygen saturation (SpO_2_) and rectal temperature (RT). * Significant difference within each group (*p* < 0.05). † Significant difference between groups (*p* < 0.05). Data were expressed as mean ± SD.

**Table 3:** Echocardiographic parameters measured in goats at baseline and at 2, 10 min, and time at which alfaxalone infusion last prevented purposeful movement (t-MIR) during anesthesia maintenance with alfaxalone alone (9.6 mg/kg/h) (ALF group) or with alfaxalone (9.6 mg/kg/h) after electroacupuncture induction (EA-ALF group).

Parameter	Group	Time Points			
		Baseline	2 min	10 min	t-MIR
**EF (%)**	ALF	84.5 ± 2.9	81.2 ± 6.7	83.4 ± 4.7	78.9 ± 7.7
	EA-ALF	80.2 ± 4.8	80.3 ± 3.5	80.5 ± 5.1	81 ± 6.1
**FS (%)**	ALF	46.4 ± 3.5	43.4 ± 7.5	43.4 ± 5.4	41 ± 6.9
	EA-ALF	42.1 ± 5.3	42 ± 2.6	42.7 ± 5.1	43.3 ± 6
**SV (mL)**	ALF	12.9 ± 2.4	10.1 ± 1.7	10 ± 1.5	9.4 ± 1.5 *^,^†
					* *p* = 0.0195
					† *p* = 0.0003
	EA-ALF	13.4 ± 2	15.1 ± 2.5 †	14.6 ± 2.3 †	14.7 ± 2.2
			† *p* = 0.0005	† *p* = 0.0015	
**CO** **(L/min)**	ALF	1.2 ± 0.2	1.2 ± 0.2	1.5 ± 0.4	1.3 ± 0.3
	EA-ALF	1.3 ± 0.1	1.5 ± 0.2	1.6 ± 0.2	1.8 ± 0.2 *
					*p* = 0.0312
**CI** **(L/min/m^2^)**	ALF	1.6 ± 0.4	1.6 ± 0.2	2 ± 0.6	1.8 ± 0.6
	EA-ALF	1.8 ± 0.3	2.2 ± 0.3	2.2 ± 0.3	2.6 ± 0.4 *^,^†
					* *p* = 0.0354
					† *p* = 0.0451

Ejection fraction (EF), fraction shortening (FS), stroke volume (SV), cardiac output (CO) and cardiac index (CI). * Significant difference within each group (*p* < 0.05). † Significant difference between groups (*p* < 0.05). Data were expressed as mean ± SD.

**Table 4:** Nociceptive threshold and Flank muscle tone measured in goats at baseline and at 2, 10 min, and time at which alfaxalone infusion last prevented purposeful movement (t-MIR) during anesthesia maintenance with alfaxalone alone (9.6 mg/kg/h) (ALF group) or with alfaxalone (9.6 mg/kg/h) after electroacupuncture induction (EA-ALF group).

Parameter	Group	Time Points			
		Baseline	2 min	10 min	t-MIR
Nociceptive threshold ^(1)^	ALF	23.8 ± 2.5	28.8 ± 7.3	33.3 ± 7 *	29.5 ± 6.3
				*p* = 0.0432	
	EA-ALF	23.6 ± 5.2	46 ± 16.2 *^,^†	53.6 ± 8.6 *^,^†	48.5 ± 9 *^,^†
			* *p* < 0.0001	*^,^† *p* < 0.0001	*^,^† *p* < 0.0001
			† *p* = 0.0001		
Flank muscle tone ^(2)^	ALF	2 (2–3)	1.5 (1–2)	2 (1–2)	2 (1–2)
			*p* = 0.0456		
	EA-ALF	2 (2–3)	1 (1–2)	1.5 (1–2)	1.5 (1–2)
			*p* = 0.0456		

* Significant difference within each group (*p* < 0.05). † Significant difference between groups (*p* < 0.05). Data were expressed as mean ± SD ^(1)^ and median (range) ^(2).^

## Data Availability

The data set used for statistical analysis is available upon reasonable request.

## Appendix A

**Table A1:** Flank muscle tone scoring system.

**Score**	**Description**
0	Complete relaxation to palpation with minimal or no tone palpable.
1	Detectable relaxation to palpation with less tone palpable.
2	Normal resting tone: flank has some elasticity to palpation.
3	Mild resistance and tone with gentle pressure on the skin and muscle.
4	Strong resistance and tone with strong pressure on the skin
